# Comparative Phytochemical Analysis of Five Species of the Genus *Arthrophytum* Schrenk (Amaranthaceae) from the Flora of Kazakhstan

**DOI:** 10.3390/metabo15120800

**Published:** 2025-12-17

**Authors:** Serikbay Ussen, Polina V. Vesselova, Gulmira M. Kudabayeva, Meruyert M. Sergazina, Mereke B. Alimzhanova

**Affiliations:** 1 Institute of Botany and Phytointroduction, Timiryazev Str. 36D/1, Almaty 050040, Kazakhstan; pol_ves@mail.ru (P.V.V.); kgm_anita@mail.ru (G.M.K.); 2 Faculty of Biology and Biotechnology, Al-Farabi Kazakh National University, 71 al-Farabi Ave., Almaty 050040, Kazakhstan; mereke.84@mail.ru; 3 Center of Physical Chemical Methods of Research and Analysis, Al-Farabi Kazakh National University, Kazakhstan, al-Farabi Ave. 71, Almaty 050040, Kazakhstan; sergazina.meruyert@gmail.com

**Keywords:** Amaranthaceae, *Arthrophytum*, gas chromatography–mass spectrometry (GC-MS), phytochemical analysis

## Abstract

Background: The genus *Arthrophytum* Schrenk (Amaranthaceae Juss.) is a relict systematic group restricted to the desert regions of northern Turan. Its species are narrowly endemic, stenotopic, and poorly studied, with virtually no available data on their phytochemical composition. Objectives: This study aimed to conduct the first comparative phytochemical analysis of five *Arthrophytum* species—*A. lehmannianum*, *A. iliense*, *A. longibracteatum*, *A. subulifolium*, and *A. betpakdalense*—to reveal their metabolite profiles and assess chemotaxonomic and functional features. Methods: Phytochemical profiling was performed using gas chromatography–mass spectrometry (GC–MS) to identify volatile and semi-volatile metabolites in the studied species. Results: GC–MS analysis revealed a predominance of terpenes in all species, along with significant contributions from fatty acids, esters, and other oxygen-containing compounds. The taxa were characterized by a rich pool of isoprenoids, including terpenes, sterols, tocopherols, and squalene, as well as lipid components of cuticular coatings such as fatty acids and long-chain alcohols. Isoprenoids dominated particularly in *A. subulifolium* and *A. longibracteatum*. *A. iliense* showed a high content of carbonyl and aromatic compounds, whereas *A. longibracteatum* and *A. lehmannianum* were distinguished by elevated levels of fatty acids and long-chain alcohols. Common metabolites—β-sitosterol, stigmasterol, vitamin E, squalene, and caryophyllene—constituted the conservative biochemical core of the genus. Conclusions: The results obtained for the first time demonstrate distinct chemotaxonomic and functional features of relict *Arthrophytum* species and highlight their potential for further research and application in the pharmaceutical, cosmetic, and aromatic industries.

## 1. Introduction

During many years of expeditionary research, we studied representatives of the Amaranthaceae family, which is systematically complex and the largest in the flora of arid ecosystems in Kazakhstan, with an emphasis on species that demonstrate a high degree of ecological adaptation to extreme environmental conditions. During field observations, particular attention was drawn to the narrowly endemic and relict genus *Arthrophytum* Schrenk (Amaranthaceae), which includes seven species [1,2]—*Arthrophytum iliense* Iljin, *A. betpakdalense* Korovin & Mironov, *A. subulifolium* Schrenk, *A. lehmannianum* Bunge, *A. korovinii* Botsch., *A. longibracteatum* Korovin, and *A. pulvinatum* Litv.—whose ranges are confined to the deserts of Central and Southern Kazakhstan.

These plants are a valuable element of ancient desert flora complexes, which have retained unique morphophysiological, anatomical, and biochemical adaptations that ensure their survival in harsh arid climates [3,4].

Despite their high adaptive potential [1,5] and ecological significance, representatives of the *Arthrophytum* genus remain virtually unstudied, including in terms of phytochemistry, due to both the complexity of species identification and the rarity of natural populations. Meanwhile, data on the composition of secondary metabolites of such species may be crucial for understanding the mechanisms of their adaptation, as well as for the search for new natural antioxidants and terpene compounds with promising biotechnological potential [6,7,8].

Phytochemical studies of the genus *Arthrophytum* have so far been extremely limited and have focused only on a few individual species. In particular, earlier investigations addressed the taxon formerly referred to as *Arthrophytum scoparium* [9], which is now placed in a separate genus and classified as *Haloxylon scoparium* Pomel [10]. In addition, the study by Dif et al. demonstrated that methanolic and ethyl acetate (medium-polarity) extracts of *Arthrophytum schmittianum* contain substantial amounts of phenolic compounds and condensed tannins, and exhibit pronounced antioxidant and antimitotic activities. At the same time, systematic interspecific comparisons of phytochemical composition across the genus are either absent or highly fragmentary [11]. The relationships between chemical profiles, taxonomy, ecological conditions (desert/semi-desert habitats), and biological activity remain insufficiently understood.

In order to identify conservative chemical compounds reflecting the adaptive characteristics of representatives of the genus, we conducted a comparative phytochemical analysis of five species of *Arthrophytum* Schrenk (Amaranthaceae)—*A. lehmannianum* Bunge, *A. iliense* Iljin, *A. longibracteatum* Korovin, *A. subulifolium* Schrenk, and *A. betpakdalense* Korovin & Mironov—using gas chromatography–mass spectrometry (GC–MS). The results obtained made it possible to characterize the metabolic profile of representatives of the genus *Arthrophytum* for the first time and to identify chemical markers of their ecological and evolutionary adaptation to desert conditions, which is important for further functional, chemotaxonomic, and applied research.

## 2. Materials and Methods

### 2.1. Geobotany Methods

**Figure 1:**
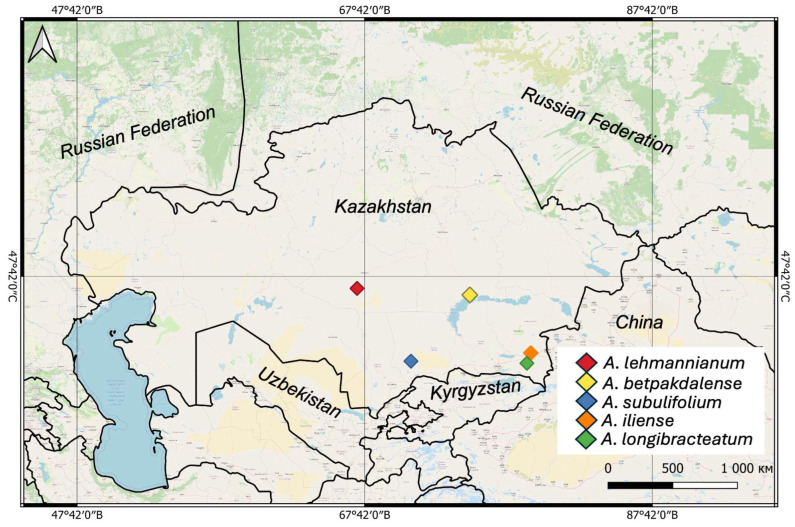
Map of plant material collection.

**Figure 2:**
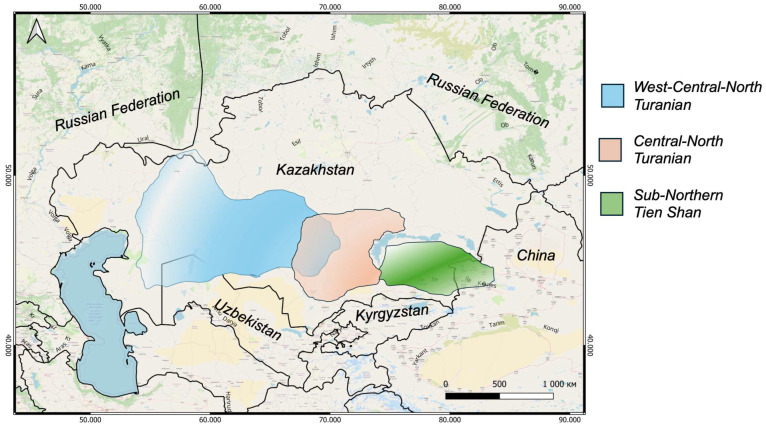
Species range map. Western-central-northern Turan species *A. lehmannianum*. Central-northern Turan species *A. betpakdalense* and *A. subulifolium*. Northern Tien-Shan species *A. iliense* and *A. longibracteatum*.

In the course of the study, classical botanical methods were used, including the route, ecological–systematic, and ecological–geographical approaches (Figure 1). The route method was used to survey natural habitats and identify areas with the growth of species of the genus *Arthrophytum* (Figure 2) and the ecological preferences of species. GPS and GIS technologies were used to record coordinates and build distribution maps [12].

### 2.2. Morphological Methods

Species of the genus *Arthrophytum* were identified using the international online databases IPNI (International Plant Names Index) and POWO (Plants of the World Online) to verify nomenclature and taxonomic status. Detailed photographs of the diagnostic structures under a stereomicroscope were taken using a Canon digital camera for later analysis and documentation. All the data obtained were systematized and used to clarify the boundaries of species and intraspecific variability.

### 2.3. Chemical Methods

The plant material was collected in the field and transported to the laboratory, where it was air-dried at room temperature (22–25 °C) in a shaded, well-ventilated environment for 10–14 days until a constant weight was reached. After drying, the samples were ground in a laboratory mill to a homogeneous fine powder (particle size < 1 mm) to ensure uniform solvent penetration during extraction.

For the extraction of metabolites, 1.0 g of powdered plant material was placed into a glass extraction flask and covered with 10 mL of an analytical-grade organic solvent (hexane or dichloromethane; purity ≥ 99%). The extraction was performed at room temperature under continuous magnetic stirring (300–400 rpm) for 24 h. To improve extraction efficiency, the mixture was vortexed for 30 s before the start of extraction and again after 12 h of maceration.

After extraction, the mixture was filtered through a paper filter, followed by a 0.45 μm PTFE membrane to remove suspended particles. The filtrate was evaporated to dryness using a rotary evaporator under reduced pressure (150–200 mbar) at a temperature not exceeding 40 °C to avoid thermal degradation of volatile components. The dry residue was then dissolved in 1 mL of the same solvent (hexane or dichloromethane), vortexed for 20 s, and transferred into an autosampler vial equipped with a low-volume insert.

GC–MS analysis was performed on an Agilent 7890A gas chromatograph (Santa Clara, CA, USA) coupled to a 5975C mass selective detector. Separation was carried out on an HP-5MS capillary column (30 m × 0.25 mm × 0.25 μm). The oven temperature program was as follows: initial temperature of 60 °C (hold 2 min), ramp at 5 °C/min to 300 °C, and final hold for 10 min. The injector temperature was 250 °C, with an injection volume of 1 μL in split mode (split ratio of 1:50). Helium (99.999% purity) served as the carrier gas at a constant flow rate of 1.0 mL/min.

The MS detector operated in electron impact ionization mode (70 eV), scanning the mass range at m/z 40–550. The ion source and quadrupole temperatures were set at 230 °C and 150 °C, respectively.

Compound identification was carried out by comparing the obtained mass spectra with NIST 14 library data (similarity index ≥ 85%) and by calculating retention indices using a series of n-alkane standards (C8–C30), followed by comparison with published reference values. Quantification was based on peak area normalization, expressing the relative content of each component as a percentage of the total ion chromatogram area. Statistical analysis of the data was performed as follows: all measurements were carried out in triplicate, and the results are expressed as mean ± standard deviation. The relative content of the components was normalized to 100% for each species to ensure accurate interspecies comparison [13,14,15].

## 3. Results

### 3.1. Distribution Analyses

During the study period, six expeditions were carried out, during which six populations of five species of the genus *Arthrophytum* (*A. lehmannianum* Bunge, *A. iliense* Iljin, *A. longibracteatum* Korovin, *A. subulifolium* Schrenk, and *A. betpakdalense* Korovin & Mironov) were described. Most species of this genus are rare, narrowly localized endemics [2], which we discovered only in the second year of our thorough search. The collected herbarium materials from different regions were scanned (Appendix A) and stored in the herbarium collection (AA) of the Institute of Botany and Phytointroduction, Almaty (Table 1). Raw material was collected from each species for further research (Figure 1).

As a result of analyzing literature data [16,17,18], herbarium materials, and our own research, it has been established that the distribution of the studied species of the genus *Arthrophytum* is characterized by pronounced regional specificity. For example, the west-central-northern Turan species *A. lehmannianum* has the most extensive range, occupying mainly the western regions of Kazakhstan and being geographically isolated from other representatives of the genus.

The central-northern Turan species *A. betpakdalense* and *A. subulifolium* are confined to the desert regions of Betpakdala and Moyinkum, where they form isolated populations adapted to arid conditions.

The north Tianshan species *A. iliense* and *A. longibracteatum* are distributed in the foothill deserts of south-eastern Kazakhstan, mainly in areas adjacent to the Tien-Shan Mountains (Figure 2).

Thus, analysis of the geographical distribution showed a clear distinction between the ranges of species according to natural climatic zones, reflecting their ecological specialization and the historical and geographical formation of the *Arthrophytum* genus flora in Kazakhstan.

### 3.2. Morphological Analysis

According to literary sources [19], representatives of the *Arthrophytum* genus are small shrubs or semi-shrubs with jointed, brittle stems and opposite awl-shaped leaves, which are sometimes poorly developed. The flowers are solitary, located in the axils of bracts similar to stem leaves; they are small and bisexual, with two bracts. The perianth is five-membered and flattened–spherical, with almost rounded, free leaves at the base; during fruiting, they acquire wing-like growths or calloused thickenings. The stamens are fused at the base into a disk, thickened at the edge, and often glandular or fringed-glandular. The ovary is two-celled, with two stigmas.

The species of the genus *Arthrophytum* are easily distinguished from each other by their external characteristics, which confirms their taxonomic independence [19].

**Figure 3:**
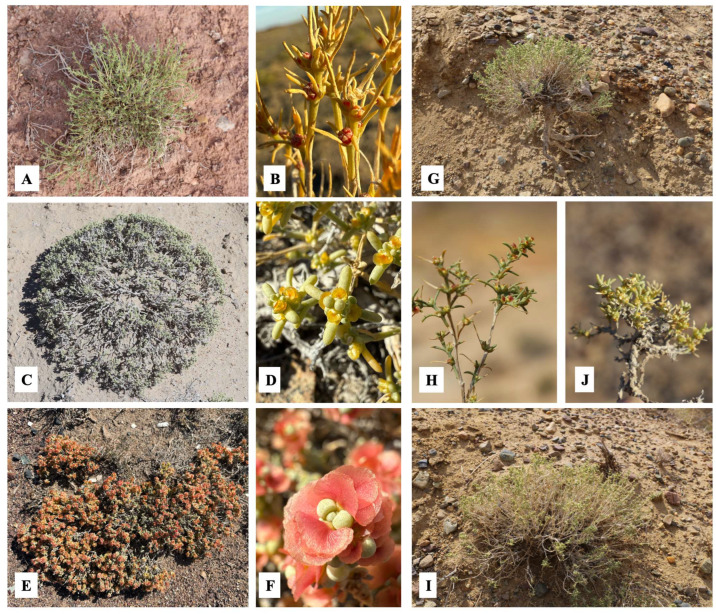
Morphological traits of *Arthrophytum* species: (**A**,**B**)—*A. subulifolium*; (**C**,**D**)—*A. lehmannianum*; (**E**,**F**)—*A. betpakdalense*; (**G**,**H**)—*A. iliense*; (**I**,**J**)—*A. longibracteatum*).

The species *A. lehmannianum* is characterized by elongated, fleshy leaves and short, spherical bracts. In the species *A. iliense* and *A. longibracteatum*, the leaves are more elongated and thin, and the bracts are elongated, and in *A. betpakdalense*, the leaves are spherical in shape. Despite their external similarity, *A. iliense* and *A. longibracteatum* have been described as separate taxa because there are minor but stable differences between them, mainly in leaf length. *A. subulifolium* is distinguished by its needle-like leaves and wingless fruits (Figure 3).

### 3.3. Phytochemical Analysis

The genus *Arthrophytum* (Amaranthaceae) includes desert and semi-desert species that have developed ecological and physiological strategies to withstand arid conditions. Chemically, such taxa are often characterized by a developed pool of isoprenoids (terpenes, sterols, and tocopherols), as well as lipid components of cuticular coatings (fatty acids and long-chain alcohols), which determine their potential antioxidant and membrane-protective activity, aromatic profile, and applied value. Despite local reports on the biological activity of individual representatives, comparable interspecies data on volatile/semi-volatile components remain limited.

The aim of this study was to conduct a comparative phytochemical analysis of five species of *Arthrophytum*—*A. lehmannianum*, *A. iliense*, *A. longibracteatum*, *A. subulifolium*, and *A. betpakdalense*—using gas chromatography–mass spectrometry (GC-MS) and to obtain the results of identified compounds. For each species, lists of components were compiled, indicating retention time, presumed identification, relative content, and standard deviation (Table A1, Table A2, Table A3, Table A4 and Table A5). The data were then unified and aggregated by chemical class, followed by normalization to 100% per species, which ensured correct interspecies comparison.

To improve clarity and interpretability, the profile is presented in two complementary visualizations: (i) a stacked bar chart showing the relative contribution of classes by type (Figure 1) and (ii) a heat map showing the distribution of classes (Figure 3). The classification has been deliberately simplified: isoprenoids (terpenes of all levels together with sterols, tocopherols, and squalene), carbonyls (aldehydes + ketones), fatty acids, alcohols, esters, n-alkanes/alkenes, epoxides, and other/unassigned (rare/ambiguous annotations). This approach allows us to simultaneously capture the overall biogenetic ‘framework’ and identify second-tier interspecies accents. The following sections present the results, their discussion, and their comparison with literature data, including an assessment of the contributions of the isoprenoid block and lipid components, as well as potential biological and applied implications.

**Figure 4:**
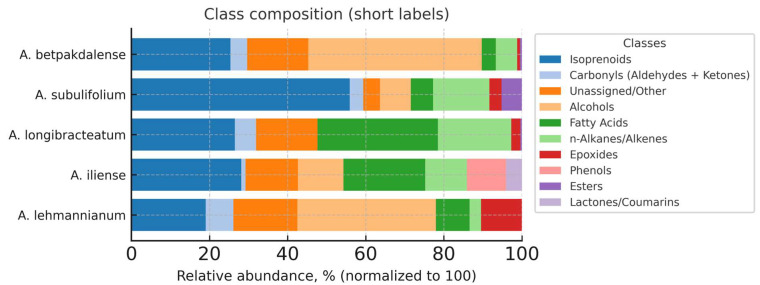
Class composition of volatile/semi-volatile compounds of five species of *Arthrophytum*.

The diagram (Figure 4) shows the relative contribution of chemical classes for five species of *Arthrophytum*, normalized to 100% for each species. Aggregated classes were used: isoprenoids (terpenes of all levels + sterols + tocopherols + squalene), carbonyls (aldehydes + ketones), and other/unassigned. Other classes were retained: fatty acids, alcohols, esters, n-alkanes/alkenes, epoxides, plasticizers/phthalates, etc. Isoprenoids are the largest block for all species, the most pronounced in *A. subulifolium* and *A. longibracteatum*, and consistently high in *A. betpakdalense* and *A. lehmannianum*; in *A. iliense*, the proportion of isoprenoids also dominates, but the profile is supplemented by aromatic/carbonyl components.

Fatty acids and conjugated long-chain alcohols confidently form the second echelon, especially in *A. longibracteatum* and A*. lehmannianum* (fatty acid–alcohol accent). Carbonyls (aldehydes + ketones) are most prominent in *A. iliense* against the background of phenolic/coumarin compounds. n-alkanes/alkenes and epoxides are present as profile modifiers (higher in *A. lehmannianum*; moderately in *A. subulifolium* and *A. betpakdalense*). Potential background/technogenic markers (plasticizers/phthalates) occur focally and do not affect the overall “skeleton” of the profile.

The dominance of isoprenoids in all five species indicates a common biogenetic nucleus of the genus: terpene derivatives, sterols, and tocopherols/squalene form a stable “protective” and structural pool (membrane stability and antioxidant protection). Interspecies differences are determined by the balance of isoprenoids with fatty acids/alcohols and the contribution of carbonyls:

*A. longibracteatum* and *A. lehmannianum*: a pronounced lipid–wax component (FA + alcohols).

*A. subulifolium*: sterol–tocopherol enrichment within isoprenoids.

*A. iliense*: an increased proportion of xarbonyls (together with phenolic/lactone components) is a possible marker of the specificity of secondary metabolism and odor profile.

*A. betpakdalense*: smooth isoprenoid pool with appreciable alcohols of C28.

Thus, the isoprenoid–sterene “framework” supports the prospect of antioxidant/dermatotropic applications; fatty acid/alcohol-focused species are potentially interesting for wax/barrier formulas, acting as an “aromatic” trace of *A. iliense* for destinations where phenolic volatiles are important.

**Figure 5:**
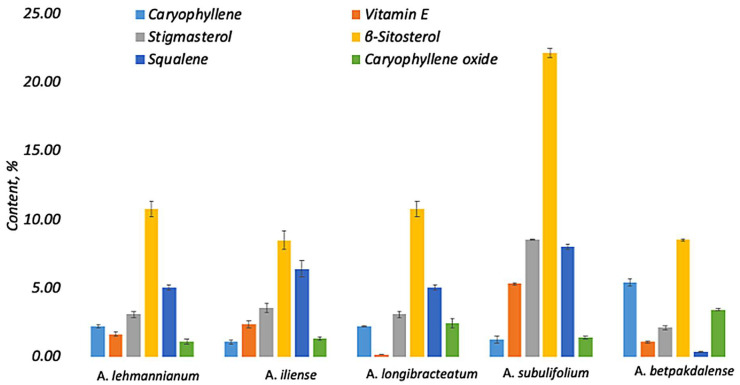
Comparison of the content (%) of total metabolites in five species of *Arthrophytum* (mean ± SD; *n* = 3).

Figure 5 shows the compounds found in all species: β-sitosterol, stigmasterol, vitamin E (tocopherol), squalene, caryophyllene, and caryophyllene oxide.

In all five species, β-sitosterol dominates, with the highest proportion observed in *A. subulifolium* (a pronounced peak), while in the other species, the contribution of this sterol is moderate. The second echelon is formed by vitamin E and squalene; their levels vary between species: vitamin E is relatively higher in *A. iliense* (and noticeable in *A. subulifolium*), while squalene is comparable in most species without a pronounced leader. Stigmasterol is consistently present at an average level, maintaining a “sterol” profile along with β-sitosterol. Caryophyllene gives a moderate contribution in all species, while caryophyllene oxide remains a minor component.

Thus, the common “framework” of the profile is set by the sterol fraction (β-sitosterol > stigmasterol); vitamin E and squalene provide a significant secondary contribution and vary between species; and terpene components (caryophyllene and its oxide) are present in all species, but mainly as minors. This configuration emphasizes the stereotyping of the basic metabolic nucleus during interspecies shifts in the “second echelon” of compounds.

**Figure 6:**
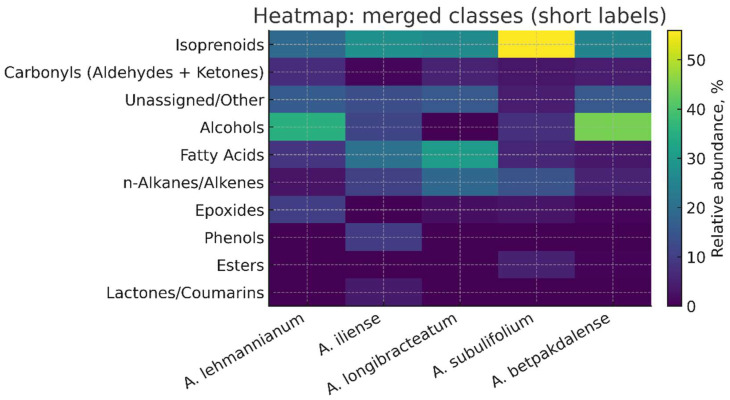
Heat map of the class profile of five species of *Arthrophytum*.

The heat map (Figure 6) visualizes the distribution of enlarged classes among five species; the scale reflects the relative share (%). The warmest (highest) values in the series of isoprenoids (terpenoids) were noted for *A. subulifolium* and *A. longibracteatum*, followed by *A. betpakdalense* and *A. lehmannianum*. *A. iliense* retains isoprenoid dominance but has additional “hot spots” in carbonyl blocks and conjugated phenolic/lactone subgroups.

### 3.4. Class Composition of Volatile Components by Arthrophytum Species

*A. lehmannianum* is dominated by terpenes; the second and third contributions are formed by fatty acids and esters, which indicates a combination of the volatile terpene fraction with a “heavier” fatty acid component and a noticeable proportion of volatile/semi-volatile esters.

In *A. iliense*, terpenoids again occupy a leading position, but esters are relatively more pronounced than in other species, highlighting the “ester” component of the profile. Oxygen-containing low-molecular-weight classes give a moderate but comparable contribution. In *A. longibracteatum*, the terpene-oriented profile also predominates, and fatty acids are consistently among the second three, increasing the “lipophilicity” of the overall picture. *A. subulifolium* is also in first place, and esters and alcohols with phenols are in the top 3, which emphasizes the more pronounced presence of oxygen-containing derivatives (potentially significant odorants). Terpenes dominate in *A. betpakdalense*, while fatty acids remain consistently among the three most abundant compounds. Aldehydes and ketones are noted among the minor classes, which may reflect more active oxidation/degradation processes or specific biogenetic pathways.

Thus, terpenes are the leading class in all five species. In terms of the relative proportion of terpenes, species form a “dense group”, but in *A. iliense* and *A. subulifolium*, the participation of oxygen-containing derivatives (esters and alcohols/phenols) is noticeably higher, which “dilutes” the proportion of purely hydrocarbon terpenes. *A. lehmannianum* and *A. longibracteatum* demonstrate a comparably increased proportion of fatty acids, forming a stable second echelon after terpenes. *A. betpakdalense* also makes a significant contribution of fatty acids with a comparable representation of minor oxygen-containing classes. *A. iliense* and *A. subulifolium* are the most “ethereal” of the five, as their ester contribution is above average for species, which is consistent with pronounced odorous notes and may indicate the specifics of secondary metabolism/raw materials. *A. subulifolium* is relatively rich in alcohols and phenoams, while in *A. betpakdalense*, it is above average in aldehydes and ketones, which is interpreted as a possible contribution of oxidative processes or specific biogenetic pathways. The balance of terpenes and oxygen-containing derivatives (esters, alcohols/phenols, and aldehydes/ketones) and the contribution of fatty acids is assessed. This distribution is consistent with the variability of secondary metabolism and can serve as a starting point for the isolation of chemomarkers at the level of individual compounds.

## 4. Discussion

Our profiles of five *Arthrophytum* species (*A. lehmannianum*, *A. iliense*, *A. longibracteatum*, *A. subulifolium*, and *A. betpakdalense*) obtained by GC–MS volatile/semi-volatile components demonstrate a stable dominance of the terpene pool with a significant participation of fatty acids and esters. This is generally consistent with the literature data on the high contribution of terpenes and lipophilic metabolites in representatives of the Amaranthaceae/Chenopodiaceae family and related genera, as well as data on the pharmacological activity of *Arthrophytum* and related taxa [9,11,20,21,22,23,24,25,26,27].

In our sets, mono- and sesquiterpenoids are the leading ones in all five species, while for *A. iliense* and *A. subulifolium*, there was a relative increase in oxygen-containing derivatives (esters and alcohols/phenols). Such a shift fits well with the studies showing pronounced antioxidant, anti-inflammatory, and other biological effects of polyphenolic/phenolic fractions and partially oxidized terpene derivatives for *A. scoparium* [11,20,21,26,28]. For example, Chao et al. demonstrated inhibition of melanogenesis by ethanol extract of *A. scoparium*, linking the effect to the presence of phenolic compounds (including coumaric/cinnamic acids and catechol), which, by our logic, correspond to the oxygen-containing block [11]. Although our instrumental focus is GC–MS (volatile/semi-volatile) and that of Chao et al. is the LC–MS phenolic range, both approaches indicate the importance of “oxygenated” components for biological activity.

In *A. lehmannianum* and *A. longibracteatum,* fatty acids consistently form a “second echelon” after terpenes. This pattern is consistent with the data for desert/semi-desert Amaranthaceae, which show a significant role of the fatty acid/wax fraction in the profiles, as well as with GC–MS observations in closely related genera (e.g., *Anabasis salsa*), and the richness of fatty acids, steroids, and other lipophilic classes [27,29]. The presence of a significant lipophilic pool may mediate antiradical and membrane-stabilizing effects, which are indirectly consistent with the antioxidant activity observed in vitro in a number of studies on *Arthrophytum* [20,21,26].

For *A. iliense* and *A. subulifolium*, we observe an increased proportion of esters, which is consistent with the “aromatic” characteristics of the profile and may correspond to more pronounced odorous notes/volatile odorants. Literature data on *Arthrophytum* and related species indicate variability of ether/oxygen-containing fractions depending on the extractant, organ, and growing conditions, including high activity of ethyl acetate fractions in *A. scoparium* and *A. schmittianum* according to antioxidant tests [9,20,22,26]. This is consistent and indicates that the “ester” shift in our samples may have functional implications for antioxidant/antiradical properties.

### 4.1. Correlation with Biological Effects from Reviews

A number of studies have linked *A. scoparium* extracts to antioxidant activity (DPPH/ABTS and β-carotene/linoleic acid), in vitro/in vivo anti-inflammatory effects, inhibition of α-glucosidase, and anti-melanogenic properties [9,20,21,24,25,26]. Our profiles indicate chemical classes that can rationally be behind some of these effects: terpene pool (including oxygenated terpenoids), esters, and phenolic-containing volatile/semi-volatile components. An increased proportion of oxygen-containing terpenes/esters in *A. iliense* and *A. subulifolium* may correlate with higher antiradical/aromatic activity noted for related fractions in the literature [9,11,20,22,26]. At the same time, it should be remembered that the polyphenol pool (catechol, chrysoeriol, etc.) “looks” at LC approaches, while our GC–MS predominantly fixates on volatile/semi-volatile approaches.

### 4.2. Ecological and Methodological Factors of Variability

The variability of class compositions can be explained not only by interspecies differences, but also by environmental conditions, organ/phenological phase, and extractant. Works on ecology and soil factors in arid biotopes show significant spatial heterogeneity and gradients of salinity, organic carbon, and available nitrogen/phosphorus, affecting plant metabolism [30,31]. *Anabasis aphylla* shows a weak or variable relationship with individual soil parameters, but a strong inverse correlation between total phenolic content and IC50 [32]. These observations support the idea that the class shifts we identified (e.g., an increase in the proportion of esters/oxygenated terpenes) may be partly environmental/population adaptation-related. Methodologically, the literature demonstrates a strong dependence of the formulation on the extractant: aqueous/boiled fractions are rich in polyphenols and tannins [20,22,26], while organic solvents and GC–MS-oriented approaches distinguish volatile/semi-volatile classes (terpenes, esters, and fatty acids) [29].

A number of sources on *Chenopodiaceae*/*Amaranthaceae* and related taxa emphasize the presence of alkaloids (including indole/β-carboline, anabasine, etc.) [27,33,34,35,36,37]. As a rule, they are more polar and thermolabile than the main volatile phase, so they often escape direct registration in GC-MS without special derivatization/extraction, rather than because of their biosynthetic absence.

Our results confirm the “terpene-centric” nature of *Arthrophytum* volatile profiles and complement existing data by showing species-specific shifts in the fractions of oxygen-containing derivatives and fatty acids. This distribution agrees well with the published pharmacological activity of *Arthrophytum* extracts and related taxa, as well as with the ecological variability of desert communities. The simultaneous use of GC-MS (volatile) and LC-MS (polyphenols/alkaloids) will further link the chemical class profile with functional effects at the level of specific molecules.

## 5. Conclusions

A study of the chemical composition of five species of the genus *Arthrophytum* (*A. lehmannianum*, *A. iliense*, *A. longibracteatum*, *A. subulifolium*, and *A. betpakdalense*), carried out by GC–MS analysis of volatile and semi-volatile compounds, made it possible to identify a stable dominance of the terpene pool with a noticeable participation of fatty acids and esters. The results obtained are consistent with the literature data for representatives of the families Amaranthaceae and Chenopodiaceae, characterized by a high content of lipophilic metabolites. For all the studied species, mono- and sesquiterpenoids are the leading components, but for *A. iliense* and *A. subulifolium*, an increase in the proportion of oxygen-containing derivatives, including esters and alcohols, was recorded, which is probably due to the increased antioxidant and aromatic activity of these taxa.

In *A. lehmannianum* and *A. longibracteatum*, there is a pronounced presence of fatty acids, which form the second most important group of compounds, which may be due to adaptation to arid conditions and protective membrane-stabilizing properties. *A. subulifolium* is characterized by the accumulation of esters, which explains the specific odor notes of the profile.

The data obtained emphasize the ecological and biochemical plasticity of the genus *Arthrophytum* and also confirm the functional role of terpenes and oxygen-containing compounds in the implementation of antioxidant and protective effects. The established regularities demonstrate species-specific differences in chemical profiles and confirm the prospects of an integrated approach using GC-MS and LC-MS for further study of phytochemical and pharmacological potential representatives of the genus *Arthrophytum*.

## Figures and Tables

**Table 1:** Populations and sampling information.

№	Species	N	E	Administrative Districts	Voucher
1	*A. lehmannianum*	47.133605	67.169902	Ulytau region	0003631
2	*A. iliense*	43.982948	79.242801	Almaty Region, along the highway towards Chundzha	0003629
3	*A. longibracteatum*	43.46725749	78.97789255	Almaty Region, along the highway towards Chundzha	0003635
4	*A. subulifolium*	43.57220281	70.91093101	Zhambyl Region, Akkol	0003622
5	*A. betpakdalense*	46.822778	75.008056	Karaganda Region, 1 km from the city of Balkhash	0003621

**Figure A1:**
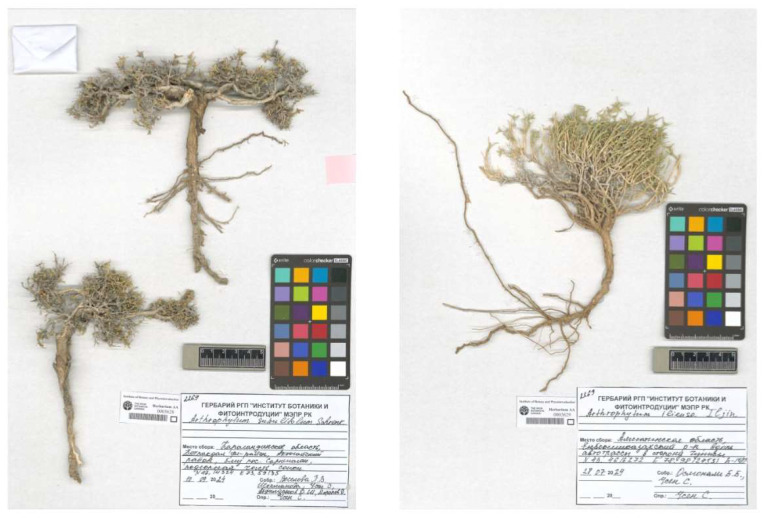
**The genus *Arthrophytum* Schrenk (Herbarium AA).**

## Data Availability

All data supporting this study’s findings are available in the main text or Appendix A and Appendix B.

## Abbreviations

DPPH	1,1-diphenyl-2-picrylhydrazyl
ABTS	2,2-azinobis 3-ethylbenzothiazoline 6-sulfonate
GC-MS	Gas Chromatography–Mass Spectrometry
LC	Liquid Chromatography
IC50	Half Maximal Inhibitory Concentration
RMS	Root Mean Square

## Appendix A

**Table A1:** GC-MS analysis of *A. lehmannianum*.

№	Retention Time, min	Compounds	Content, %	RMS
1	18.24	Caryophyllene	0.91	0.11
2	18.95	4-Methyl-1-(acetoxy)benzene	3.23	0.20
3	23.98	Caryophyllene oxide	1.10	0.20
4	24.61	Guanosine	3.93	0.15
5	26.01	D-Glucopyranose, 1,6-anhydro-	0.96	0.29
6	29.20	Mome inositol	2.81	0.19
7	30.40	n-Hexadecanoic acid	6.70	0.20
8	32.69	Phytol	1.00	0.26
9	33.21	Dimethyl 6-(trimethylsilyl)pyrazolo [1,5-a]pyridine-2,3-dicarboxylate	0.37	0.11
10	34.10	cis-Vaccenic acid	1.19	0.28
11	34.42	9,12-Octadecadienoic acid	0.76	0.17
12	34.80	Oxirane, hexadecyl-	0.52	0.17
13	35.80	1-Octadecanol	1.53	0.16
14	36.49	Tetradecanal	0.37	0.05
15	37.26	Octacosane	0.95	0.22
16	38.11	Hexadecanal	0.78	0.07
17	39.04	Behenic alcohol	3.16	0.25
18	40.30	Octacosane	1.08	0.18
19	41.17	Octadecanal	2.12	0.17
20	42.06	Lignoceric alcohol	9.33	0.29
21	43.13	Hexatriacontane	1.33	0.32
22	44.03	Octadecanal	3.83	0.30
23	44.22	Squalene	4.62	0.16
24	44.86	n-Tetracosanol-1	10.57	0.62
25	45.46	1,6,10,14-Hexadecatetraen-3-ol, 3,7,11,15-tetramethyl-	1.02	0.30
26	45.77	Tetratetracontane	0.89	0.28
27	46.11	1-docosanol	0.38	0.04
28	46.70	Oxirane, heptadecyl-	9.94	0.75
29	47.49	Octacosanol	10.51	0.77
30	49.19	1,30-Triacontanediol	2.72	0.20
31	50.08	Vitamin E	2.97	0.16
32	52.59	Stigmasterol	2.03	0.24
33	53.57	β-Sitosterol	6.39	0.44

**Table A2:** GC-MS analysis of *A. iliense*.

№	Retention Time, min	Compounds	Content, %	RMS
1	18.23	Caryophyllene	1.22	0.11
2	18.93	2-Methoxy-4-vinylphenol	8.08	0.06
3	21.10	Phenol, 2,6-dimethoxy-	1.10	0.06
4	23.98	Caryophyllene oxide	1.57	0.09
5	24.14	2-methoxy-4-(n-propyl)phenol v	0.82	0.10
6	24.58	Coumarin	4.10	0.06
7	24.81	Ethanone, 1-(4-hydroxy-3-methoxyphenyl)-	1.51	0.10
8	25.81	2-Propanone, 1-(4-hydroxy-3-methoxyphenyl)-	0.81	0.10
9	25.89	3,7,11,15-Tetramethyl-2-hexadecen-1-ol	0.60	0.06
10	26.00	D-Allose	1.40	0.10
11	26.19	2H-1-Benzopyran-3,4-diol, 2-(3,4-dimethoxyphenyl)-3,4-dihydro-6-methyl-, (2α,3α,4α)-	0.42	0.05
12	26.41	Tetradecanoic acid	1.08	0.07
13	26.96	2(1H)-Pyridinone, 1-cyclohexyl-3,4,5,6-tetramethyl-	0.94	0.11
14	27.40	2-Pentadecanone, 6,10,14-trimethyl-	0.56	0.15
15	30.39	n-Hexadecanoic acid	9.54	0.41
16	34.10	cis-Vaccenic acid	6.26	0.30
17	34.42	9,12-Octadecadienoic acid	4.02	0.20
18	35.79	n-Heptadecanol-1	2.28	0.15
19	37.25	Hexacosane	2.49	0.06
20	38.11	Octadecanal	0.53	0.05
21	38.32	4,8,12,16-Tetramethylheptadecan-4-olide	0.91	0.11
22	39.04	Behenic alcohol	3.42	0.25
23	40.29	Hexacosane	2.06	0.25
24	42.04	n-Tetracosanol-1	8.23	0.13
25	43.12	Octacosane	3.19	0.10
26	44.20	Squalene	7.41	0.15
27	45.45	1,6,10,14-Hexadecatetraen-3-ol, 3,7,11,15-tetramethyl-	2.58	0.20
28	45.77	Hentriacontane	2.95	0.10
29	46.18	9,19-Cycloergost-24(28)-en-3-ol, 4,14-dimethyl-, acetate, (3β,4α,5α)-	1.29	0.20
30	46.50	1,6,10,14,18,22-Tetracosahexaen-3-ol, 2,6,10,15,19,23-hexamethyl-	1.97	0.40
31	50.07	Vitamin E	2.73	0.15
32	52.57	Stigmasterol	4.05	0.03
33	53.55	β-Sitosterol	9.87	0.19

**Table A3:** GC-MS analysis of *A. longibracteatum*.

№	Retention Time, min	Compounds	Content, %	RMS
1	18.23	Caryophyllene	2.23	0.06
2	19.02	Ethanone, 1-(2-hydroxy-5-methylphenyl)-	1.34	0.16
3	23.98	Caryophyllene oxide	2.45	0.31
4	24.57	Sucrose	9.78	0.25
5	25.90	E-6-Octadecen-1-ol acetate	0.41	0.09
6	27.40	2-Pentadecanone, 6,10,14-trimethyl-	1.21	0.09
7	28.83	n-Heptadecanol-1	0.41	0.09
8	30.38	n-Hexadecanoic acid	16.77	0.72
9	32.52	Trichloroacetic acid, pentadecyl ester	0.42	0.07
10	32.68	Phytol	1.29	0.12
11	32.96	9-Octadecenoic acid, methyl ester	0.48	0.04
12	33.51	Phthalic acid, 6-ethyl-3-octyl butyl ester	0.41	0.08
13	34.10	Oleic Acid	7.05	0.22
14	34.42	9,12-Octadecadienoic acid	5.73	0.21
15	34.79	Tetradecanal	1.20	0.10
16	35.79	n-Heptadecanol-1	2.91	0.20
17	37.25	Heptadecane	1.63	0.16
18	38.10	Pentadecanal-	1.41	0.10
19	38.32	4,8,12,16-Tetramethylheptadecan-4-olide	1.24	0.15
20	39.04	1-Nonadecene	5.29	0.10
21	40.29	Heptadecane	1.51	0.08
22	41.17	Tetradecanal	1.63	0.11
23	42.05	1-Docosene	7.30	0.09
24	43.12	Hentriacontane	1.83	0.06
25	44.20	Squalene	5.04	0.19
26	45.76	Tetratetracontane	1.22	0.07
27	46.49	Oxirane, 2,2-dimethyl-3-(3,7,12,16,20-pentamethyl-3,7,11,15,19-heneicosapentaenyl)-	2.33	0.15
28	50.06	Vitamin E	1.64	0.15
29	52.58	Stigmasterol	3.09	0.21
30	53.55	β-Sitosterol	10.75	0.58

**Table A4:** GC-MS analysis of *A. subulifolium*.

№	Retention Time, min	Compounds	Content, %	RMS
1	18.24	Caryophyllene	1.28	0.25
2	23.98	Caryophyllene oxide	1.39	0.09
3	27.42	2-Pentadecanone, 6,10,14-trimethyl-	1.26	0.15
4	32.27	7,9-Di-tert-butyl-1-oxaspiro(4,5)deca-6,9-diene-2,8-dione	1.09	0.20
5	33.20	Indazol-4-one, 3,6,6-trimethyl-1-phthalazin-1-yl-1,5,6,7-tetrahydro-	1.96	0.12
6	33.53	Phthalic acid, butyl isohexyl ester	0.69	0.10
7	35.80	1-Eicosanol	2.49	0.26
8	37.26	Heptadecane	3.25	0.15
9	38.11	Tetradecanal	1.14	0.11
10	38.33	4,8,12,16-Tetramethylheptadecan-4-olide	1.26	0.15
11	38.37	Hexanedioic acid, bis(2-ethylhexyl) ester	1.03	0.16
12	39.05	1-Heneicosyl formate	5.24	0.07
13	40.29	Octacosane	3.82	0.27
14	41.17	Hexadecanal	1.04	0.15
15	42.05	1-Eicosanol	5.36	0.15
16	42.16	1,2-Benzenedicarboxylic acid, diisooctyl ester	4.00	0.10
17	43.12	Hentriacontane	4.31	0.12
18	44.20	Squalene	8.00	0.20
19	45.76	Heneicosane	3.03	0.11
20	46.49	Oxirane, 2,2-dimethyl-3-(3,7,12,16,20-pentamethyl-3,7,11,15,19-heneicosapentaenyl)-	3.11	0.10
21	50.07	Vitamin E	5.31	0.08
22	52.58	Stigmasterol	8.52	0.07
23	53.55	β-Sitosterol	22.13	0.32
24	56.60	Stigmasta-3,5-dien-7-one	5.16	0.15
25	57.37	Stigmast-4-en-3-one	4.14	0.14

**Table A5:** GC-MS analysis of *A. betpakdalense*.

№	Retention Time, min	Compounds	Content, %	RMS
1	13.36	2,6-Octadienal, 3,7-dimethyl-	0.82	0.07
2	14.53	Benzenemethanol, α,α,4-trimethyl-	2.01	0.09
3	15.41	Bicyclo [3.1.1]hept-3-en-2-one, 4,6,6-trimethyl-	1.53	0.06
4	16.51	2-Cyclohexen-1-one, 3-methyl-6-(1-methylethyl)-	1.83	0.06
5	18.11	Ylangene	0.32	0.07
6	18.23	Caryophyllene	5.41	0.27
7	19.74	2H-Inden-2-one, 1,4,5,6,7,7a-hexahydro-7a-methyl-, (S)-	2.80	0.20
8	20.20	1,6-Cyclodecadiene, 1-methyl-5-methylene-8-(1-methylethyl)-, [s-(E,E)]-	0.69	0.02
9	20.42	3-Cyclopenten-1-one, 2-hydroxy-3-(3-methyl-2-butenyl)-	1.42	0.07
10	23.84	1H-Cycloprop[e]azulen-7-ol, decahydro-1,1,7-trimethyl-4-methylene-, [1ar-(1aα,4aα,7β,7aβ,7bα)]-	1.21	0.09
11	23.97	Caryophyllene oxide	3.42	0.07
12	24.54	Sucrose	1.85	0.13
13	25.90	3,7,11,15-Tetramethyl-2-hexadecen-1-ol	1.21	0.08
14	27.40	2-Pentadecanone, 6,10,14-trimethyl-	0.51	0.10
15	30.43	n-Hexadecanoic acid	3.35	0.15
16	32.26	7,9-Di-tert-butyl-1-oxaspiro(4,5)deca-6,9-diene-2,8-dione	0.52	0.11
17	32.52	1-Nonadecene	0.21	0.02
18	32.68	Phytol	0.51	0.10
19	33.52	Phthalic acid, butyl cycloheptyl ester	0.27	0.03
20	34.80	Hexadecanal	0.32	0.03
21	35.78	Behenic alcohol	5.11	0.20
22	37.26	Heneicosane	1.17	0.15
23	38.32	4,8,12,16-Tetramethylheptadecan-4-olide	0.89	0.04
24	38.80	Tetradecane, 2,6,10-trimethyl-	0.41	0.08
25	39.03	Behenic alcohol	4.19	0.09
26	40.29	Octacosane	1.65	0.15
27	41.17	Tetradecanal	0.44	0.06
28	42.04	n-Tetracosanol-1	9.25	0.17
29	43.11	Hentriacontane	1.11	0.10
30	43.45	1-Docosanol, acetate	0.64	0.07
31	44.02	Oxirane, hexadecyl-	0.74	0.05
32	44.20	Squalene	0.37	0.05
33	44.85	1-Octacosanol	15.53	0.15
34	45.76	Tetratetracontane	1.30	0.10
35	46.11	Triacontyl acetate	0.50	0.01
36	46.67	Hexadecanal	1.50	0.10
37	47.47	1-Octacosanol	9.69	0.19
38	50.06	Vitamin E	1.08	0.07
39	52.57	Stigmasterol	2.14	0.15
40	53.56	β-Sitosterol	8.50	0.10
41	55.11	Stigmast-7-en-3-ol, (3β,5α,24S)-	1.42	0.14
42	56.60	Stigmasta-3,5-dien-7-one	2.17	0.16
